# Uncertain significance and molecular insights of *CPLANE1* variants in prenatal diagnosis of Joubert syndrome: a case report

**DOI:** 10.1186/s12884-024-07052-3

**Published:** 2024-12-26

**Authors:** Si-Xiu Li, Leiting Chen, Chen Deng, Dongmei Tang, Jing Zhang, Wen-Guang Hu, Yu Hu, Hua Lai, Xiao Yang

**Affiliations:** 1 Department of Pediatric Neurology, School of Medicine, Chengdu Women’s and Children’s Central Hospital, University of Electronic Science and Technology of China, Chengdu, China; 2 Institute of Electronic and Information Engineering of UESTC in Guangdong, Dongguan, Guangdong China; 3 Department of Radiology, Chengdu Women’s and Children’s Central Hospital, School of Medicine, University of Electronic Science and Technology of China, Chengdu, China; 4 Department of Obstetrics, School of Medicine, Chengdu Women’s and Children’s Central Hospital, University of Electronic Science and Technology of China, Chengdu, China; 5 Department of Prenatal Diagnosis, Chengdu Women’s and Children’s Central Hospital, School of Medicine, University of Electronic Science and Technology of China, Chengdu, China

**Keywords:** Prenatal diagnosis, Variants of uncertain significance, Molecular effects, Gene expression, *CPLANE1* gene, Joubert syndrome

## Abstract

**Background:**

Prenatal whole exome sequencing (WES) is becoming an increasingly used diagnostic tool for fetuses with structural anomalies. However, the identification of variants of uncertain significance (VUS) in clinically relevant genes can significantly complicate prenatal diagnosis and genetic counseling.

**Case presentation:**

A fetus conceived through in vitro fertilization at the third attempt presented with polydactyly and molar tooth sign at 24 + 6 weeks of gestation. Trio-based WES was performed on both parents and the affected fetus, revealing a pair of compound heterozygous *CPLANE1* variants (c.4646 A > T/p.Glu1549Val and c.1233 C > A/p.Tyr411*) potentially associated with Joubert syndrome. According to the ACMG guidelines, one of the biallelic variants was classified as VUS, and the other as pathogenic. However, these variants had no allele frequencies in the general population. The p.Tyr411* variant was classified as deleterious, while the p.Glu1549Val variant was located in highly conserved residues, was predicted to be damaging by in silico tools, and altered hydrogen bonding. Furthermore, *CPLANE1* expression was highest in the brain during the embryonic and fetal stages. These findings provide additional support for the association between *CPLANE1* variants in this fetus and Joubert syndrome. Thus, the most likely diagnosis was Joubert syndrome, and after careful consideration, the couple decided to terminate the pregnancy.

**Conclusion:**

The expression patterns of *CPLANE1* and the molecular effects of the variants may provide further evidence supporting the potential for prenatal diagnosis of Joubert syndrome in the case of biallelic VUS and pathogenic variant. This study suggests that molecular insights may play a role in interpreting VUS in clinically relevant prenatal genes for prenatal diagnosis.

**Supplementary Information:**

The online version contains supplementary material available at 10.1186/s12884-024-07052-3.

## Background

Fetal structural anomalies are observed in 2–4% of pregnancies, resulting in increased infant morbidity, mortality, and intangible suffering for families [1, 2]. Accurate and timely prenatal diagnosis is crucial for informed reproductive decision-making [1]. More recently, whole exome sequencing (WES) has been become increasingly used for prenatal diagnosis, with diagnostic yields ranging from 8.5 to 35% [3]. However, variants of uncertain significance (VUS) in primary findings, incidental findings unrelated to the clinical indication, and secondary findings of known disease genes, pose challenges to clinical practice and genetic counseling in prenatal diagnosis [4–6]. In particular, VUS identified in clinically relevant genes may affect current and future pregnancies [7]. These uncertainties may also cause parental anxiety without providing immediate benefits for decision-making [8], underscoring the need for further evidence to clarify the associations between the variants and the fetal phenotype.

Biallelic variants in the *CPLANE1* gene (OMIM* 614571) have been reported to cause Joubert syndrome (JS, OMIM# 614615), a rare disorder characterized by a peculiar cerebellar and brainstem malformations known as molar tooth sign (MTS) [9–11]. In addition, *CPLANE1* variants have also been identified in patients with Orofaciodigital syndrome (OFD) VI (OMIM# 277170), which overlaps with JS for peculiar cerebellar and brainstem malformations but has additional key features, including tongue hamartomas and/or frenulae, upper lip notch, polydactyly, and hypothalamic hamartoma. Therefore, OFD VI is also classified as JS with oral-facial-digital defects (JS-OFD) [9–11]. Prenatal diagnosis of these disorders is challenging due to phenotypic heterogeneity, limited availability of fetal phenotypes, and potential evolution of phenotypes over time. All documented prenatal cases were identified by postmortem examination for phenotypic assessment, WES or targeted exome sequencing of aborted fetal tissue, and/or functional studies [9–17]. No reports of *CPLANE1* variants in prenatal cases with JS or OFD VI have been identified through WES of fetal amniotic fluid prior to pregnancy termination.

In this study, we identified compound heterozygous *CPLANE1* variants in the amniotic fluid of a fetus with polydactyly and MTS. The biallelic variants were classified as one VUS and one pathogenic variant. Further investigation of the expression characteristics of the *CPLANE1* gene and the molecular effects of these variants may help in the interpretation of the VUS and provide additional evidence for the potential of prenatal JS diagnosis.

## Case presentation

**Fig. 1:**
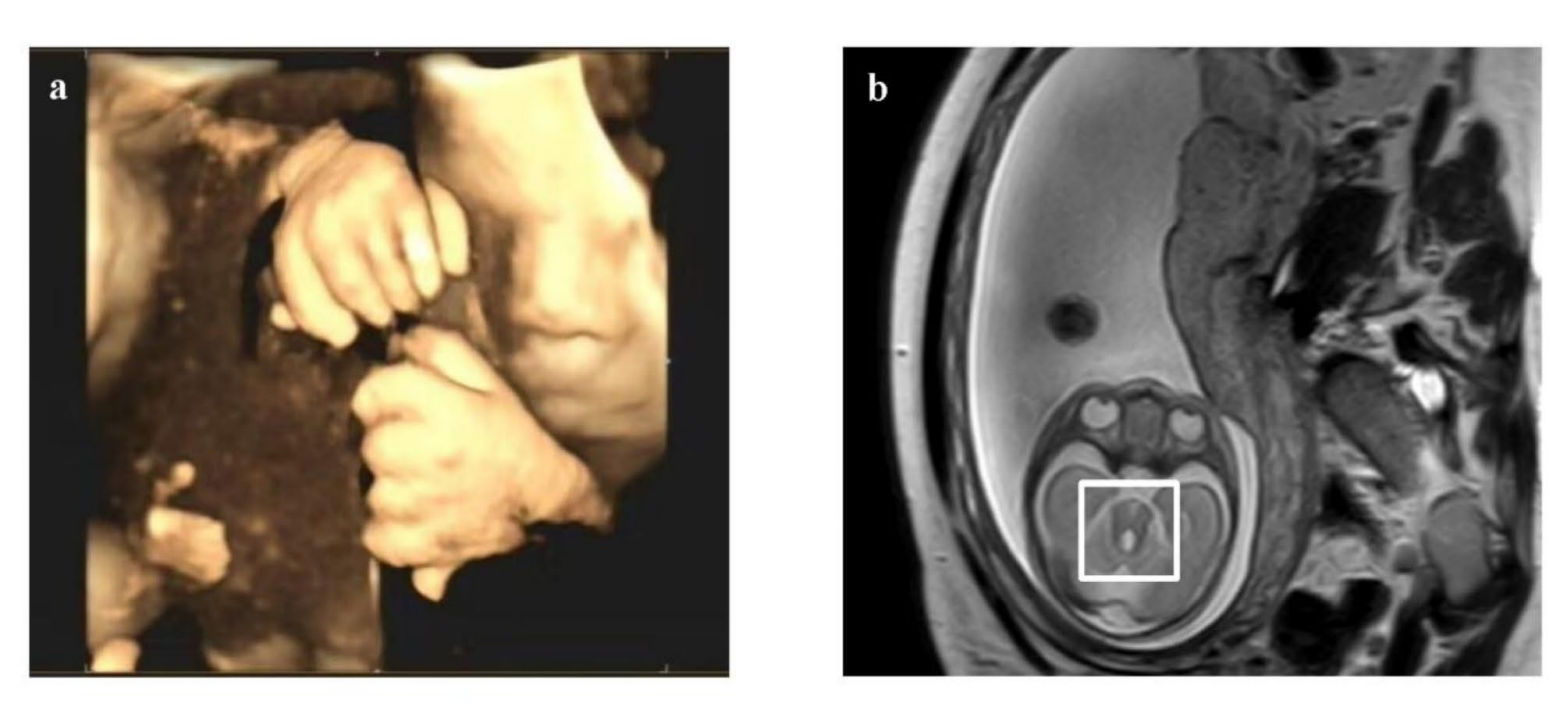
Imaging manifestations in *CPLANE1* variants as potential Joubert syndrome. (**a**) Polydactyly of both hands of the fetus on ultrasound at 33 + 1 weeks of gestation before abortion. (**b**) Partial agenesis of the vermis of the cerebellum and molar tooth signs on magnetic resonance imaging of the fetus at 25 + 1 weeks of gestation

At 24 + 6 weeks of gestation, ultrasound revealed polydactyly and possible cerebellar hypoplasia in a fetus conceived through the couple’s third in vitro fertilization (IVF) cycle after two previous unsuccessful attempts (Fig. 1a). Subsequent magnetic resonance imaging confirmed partial agenesis of the cerebellar vermis and the MTS (Fig. 1b). Trio-based WES was performed to determine the underlying cause of the observed anomalies.

Genomic DNA was extracted from fetal amniotic fluid and parental peripheral blood. Target genes were captured by probe hybridization and enriched via the IDT xGen Exome Research Panel. Sequence reads were aligned to the GRCh38/hg38 reference genome, and variant annotation was conducted via ANNOVAR software. Pathogenic variants were screened for their presence in exonic regions, nonsynonymous mutations, and frequency of less than 5% in databases such as ExAC, 1,000 Genomes, and gnomAD. Further evaluation of variants was conducted via databases including dbSNP, OMIM, HGMD, and ClinVar. Variant pathogenicity was assessed according to ACMG guidelines and confirmed through Sanger sequencing.

**Fig. 2:**
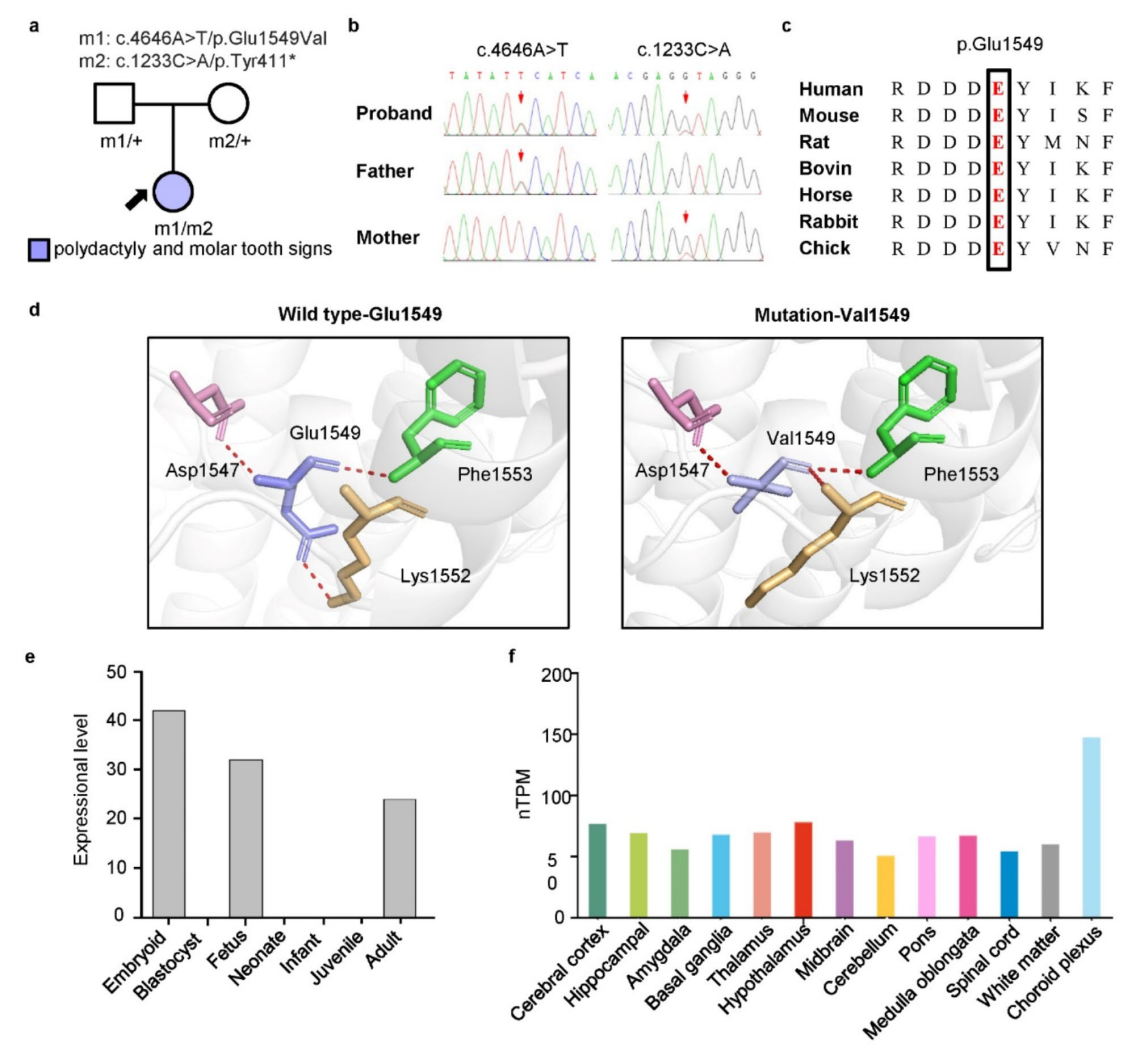
Analysis of *CPLANE1* variants and expression patterns. (**a**) Pedigrees of the case with the *CPLANE1* variant and the phenotypes. (**b**) DNA sequence chromatograms of the *CPLANE1* variants. The arrows indicate the positions of the variants. (**c**) Amino acid sequence alignment of the missense variant with protein substitutions. Glu1549 is highly conserved across species. (**d**) Hydrogen bonding changes of the p.Glu1549Val variant from the present study. (**e**) Expression pattern of *CPLANE1* in life stages, data from the UniGene database. (**f**) Expression level of *CPLANE1* in the human brain, data from the Human Protein ATLAS database. nTPM, normalized transcripts per million

A pair of compound heterozygous missense and truncation variants were identified in the *CPLANE1* gene (Fig. 2a, b, transcript NM_023073.3). According to the ACMG guidelines, the p.Glu1549Val variant is classified as a VUS (PM3 + PM2 + PP3 + PP4), while the p.Tyr411* variant is classified as pathogenic (PVS1 + PM2 + PP4) [1, 18]. Notably, the *CPLANE1* gene is primarily expressed during the embryonic and fetal stages, with the highest expression in the brain (Fig. 2e, f). Both variants were absent in the gnomAD database. Amino acid sequence alignment revealed that the p.Glu1549Val variant was located at highly conserved residues across mammals species (Fig. 2c). In addition, the p.Glu1549Val variant was predicted to be damaging by in silico prediction tools and altered hydrogen bonding with surrounding amino acids, as analyzed via the AlphaFold web tool and the PyMOL system (Table 1; Fig. 2d). Based on the clinical, genetic, and molecular findings, JS was the most likely prenatal diagnosis. After careful consideration, the couple eventually decided to terminate the pregnancy.

**Table 1:** Prediction results of *CPLANE1* variant by in silico tools

Variants (NM_023073.4)	Protein change	SIFT	FATHMM_MKL	CADD	REVEL	PROVEAN	Mutation-Taster	GERP++	GenoCanyon	M-CAP	phyloP	SiPhy
c.4646 A > T	p.Glu1549Val	D (0.002)	D (0.983)	D (32)	D (0.411)	D (-3.12)	DC (1.0)	C (5.39)	D (1.0)	D (0.091)	C (7.096)	C (15.403)

Abbreviations: C, conserved; CADD, combined annotation dependent depletion; D, damaging; DC, disease causing; FATHMM_MKL, Functional Analysis through Hidden Markov Models; GenoCanyon, Genomic Curation of Non-Coding Variants; GERP, Genomic Evolutionary Rate Profiling; M-CAP, Mendelian Clinically Applicable Pathogenicity; phyloP, Phylogenetic *P*-values; PROVEN, Protein Variation Effect Analyzer; REVEL, Rare Exome Variant Ensemble Learner; SiPhy, Selection on Integrated Phylogenetic Likelihoods; SIFT, Sorting Intolerant From Tolerant

## Discussion

The *CPLANE1* gene (also known as C5orf42) plays a crucial role in ciliogenesis and planar polarity. It is expressed primarily during the embryonic and fetal stages, particularly in the brain. In mice, homozygous deletion of cplane1 results in preweaning lethality, highlighting its importance in early development. The probability of a transcript falling into the distribution of recessive genes (pRec) is 1.00, indicating that CPLANE1 is highly intolerant to recessive loss-of-function mutations [19]. Thus, the compound heterozygous *CPLANE1* variants identified in this study are potentially loss-of-function and may be deleterious to the fetus.

Clinically, biallelic variants in *CPLANE1* have been reported to cause JS or OFD VI, which is also classified as JS-OFD [9–11]. Cases documented in the prenatal period, exhibited a range of phenotypic manifestations, including the molar tooth sign, cerebellar dysplasia, encephalocele, hydrocephalus, Blake’s fossa cyst, and Dandy-Walker malformation [9–17]. Fetal diagnosis is typically confirmed by postpartum examination of the aborted fetus for additional phenotypes, WES or targeted exome sequencing of aborted tissue, and even by functional studies (Supplementary Table 1) [9–17, 20]. Due to the limited availability of fetal phenotypes and clinical heterogeneity, definitive prenatal diagnosis based on imaging alone can be challenging, highlighting the importance of WES for prenatal diagnosis.

However, the presence of VUS associated with clinical indications in prenatal WES, particularly in cases of VUS in “trans” with a pathogenic variant, may complicate genetic counseling for fetal abnormalities and require additional support for couples compared with those with a definitive diagnosis [5, 6, 21]. Given that the couple had already had two unsuccessful IVF cycles, the decision to terminate a pregnancy should be approached with caution. In this case, the biallelic variants inherited from the asymptomatic parents were consistent with a classical recessive inheritance pattern and were not present in the gnomAD database. In addition to the truncation variant, another conserved missense variant was predicted to be deleterious by in silico tools and altered hydrogen bonding with surrounding amino acids. Besides the function of the gene and the highest expression levels in the brain during the embryonic and fetal stages, these findings further support the potential pathogenicity of the biallelic variants in *CPLANE1* and strengthen the likelihood of the prenatal diagnosis of JS.

This report has several limitations. First, the absence of a postmortem examination made it impossible to determine whether this condition was OFD VI or JS-OFD, as there was no evidence of oral, facial, or hypothalamic hamartoma features. Second, further validation of gene expression patterns and molecular effects of variants in the prenatal diagnosis of VUS cases is needed in a larger cohort of fetal samples. Third, the functional consequences of the variants were not investigated, which are essential for establishing a definitive prenatal diagnosis.

In conclusion, the expression patterns of *CPLANE1* across different organs and life stages, together with the molecular effects of the variants on protein structure, may provide further evidence supporting the potential for prenatal diagnosis of JS in the case of biallelic VUS and pathogenic variant. This study suggests that molecular insights may play a role in the interpretation of VUS in clinically relevant genes for prenatal cases.

## Electronic Supplementary Material

Below is the link to the electronic supplementary material.

Supplementary Material 1

## Data Availability

All data generated or analyzed during this study are available in the published article.

## Abbreviations

IVF: In vitro fertilization
JS: Joubert syndrome
JS-OFD: Joubert syndrome with oro-facial-digital defects
MTS: Molar tooth sign
OFD: Orofaciodigital syndrome
VUS: Variants of uncertain significance
WES: Whole-exome sequencing

## References

[1] Monaghan KG, Leach NT, Pekarek D, Prasad P, Rose NC. The use of fetal exome sequencing in prenatal diagnosis: a points to consider document of the American College of Medical Genetics and Genomics (ACMG). Genet Med. 2020;22:675–80. 31911674 10.1038/s41436-019-0731-7

[2] Calzolari E, Barisic I, Loane M, Morris J, Wellesley D, Dolk H, et al. Epidemiology of multiple congenital anomalies in Europe: A EUROCAT population-based registry study. Birt Defects Res Clin Mol Teratol. 2014;100:270–6. 10.1002/bdra.23240 24723551

[3] Guadagnolo D, Mastromoro G, Di Palma F, Pizzuti A, Marchionni E. Prenatal Exome Sequencing: Background, Current Practice and Future Perspectives—A. Syst Rev Diagnostics. 2021;11:224. 10.3390/diagnostics11020224 PMC7913004 33540854

[4] Abulí A, Antolín E, Borrell A, Garcia M, Santiago FG, Manjón IG, et al. Guidelines for NGS procedures applied to prenatal diagnosis by the Spanish Society of Gynecology and Obstetrics and the Spanish Association of Prenatal Diagnosis. J Med Genet. 2024;61:727–33. 38834294 10.1136/jmg-2024-109878

[5] Best S, Wou K, Vora N, Van Der Veyver IB, Wapner R, Chitty LS. Promises, pitfalls and practicalities of prenatal whole exome sequencing. Prenat Diagn. 2018;38:10–9. 28654730 10.1002/pd.5102 PMC5745303

[6] Westerfield L, Darilek S, Van Den Veyver I. Counseling Challenges with Variants of Uncertain Significance and Incidental Findings in Prenatal Genetic Screening and Diagnosis. J Clin Med. 2014;3:1018–32. 26237491 10.3390/jcm3031018 PMC4449641

[7] Mellis R. Next generation sequencing and the impact on prenatal diagnosis. Expert Rev Mol Diagn. 2018;18:689–99. 29962246 10.1080/14737159.2018.1493924

[8] Ferretti L, Mellis R, Chitty LS. Update on the use of exome sequencing in the diagnosis of fetal abnormalities. Eur J Med Genet. 2019;62:103663. 31085342 10.1016/j.ejmg.2019.05.002

[9] Lopez E, Thauvin-Robinet C, Reversade B, Khartoufi NE, Devisme L, Holder M, et al. C5orf42 is the major gene responsible for OFD syndrome type VI. Hum Genet. 2014;133:367–77. 24178751 10.1007/s00439-013-1385-1

[10] Dordoni C, Prefumo F, Iascone M, Pinelli L, Palumbo G, Bondioni MP, et al. Prenatal findings in oral-facial‐digital syndrome type VI: Report of three cases and literature review. Prenat Diagn. 2019;39:652–5. 31158925 10.1002/pd.5494

[11] Xiang J, Zhang L, Jiang W, Zhang Q, Wang T, Li H, et al. Prenatal Diagnosis and Genetic Analysis of a Fetus with Joubert Syndrome. BioMed Res Int. 2018;2018:7202168. 29955609 10.1155/2018/7202168 PMC6000882

[12] Wentzensen IM, Johnston JJ, Keppler-Noreuil K, Acrich K, David K, Johnson KD, et al. Exome sequencing identifies novel mutations in C5orf42 in patients with Joubert syndrome with oral–facial–digital anomalies. Hum Genome Var. 2015;2:15045. 27081551 10.1038/hgv.2015.45 PMC4785546

[13] Bonnard C, Shboul M, Tonekaboni SH, Ng AYJ, Tohari S, Ghosh K, et al. Novel mutations in the ciliopathy-associated gene CPLANE1 (C5orf42) cause OFD syndrome type VI rather than Joubert syndrome. Eur J Med Genet. 2018;61:585–95. 29605658 10.1016/j.ejmg.2018.03.012

[14] Zhu H, Chen W, Ren H, Zhang Y, Niu Y, Wu D, et al. Non-classic splicing mutation in the CPLANE1 (C5orf42) gene cause Joubert syndrome in a fetus with severe craniocerebral dysplasia. Eur J Med Genet. 2021;64:104212. 33794348 10.1016/j.ejmg.2021.104212

[15] Liu Y, Wang H, Jin X, Shao Q, Pan Q. Molecular Diagnosis and Prenatal Phenotype Analysis of Eight Fetuses With Ciliopathies. Front Genet. 2021;12:705808. 34675960 10.3389/fgene.2021.705808 PMC8523853

[16] Qian W, Liu X, Wang Z, Xu Y, Zhang J, Li H, et al. Whole-exome sequencing identified novel variants in CPLANE1 that causes oral‐facial‐digital syndrome VI by inducing primary cilia abnormality. J Cell Mol Med. 2022;26:3213–22. 35582950 10.1111/jcmm.17326 PMC9170817

[17] Qin Y, Yao Y, Liu N, Wang B, Liu L, Li H, et al. Prenatal whole-exome sequencing for fetal structural anomalies: a retrospective analysis of 145 Chinese cases. BMC Med Genomics. 2023;16:262. 37880672 10.1186/s12920-023-01697-3 PMC10601195

[18] Richards S, Aziz N, Bale S, Bick D, Das S, Gastier-Foster J, et al. Standards and guidelines for the interpretation of sequence variants: a joint consensus recommendation of the American College of Medical Genetics and Genomics and the Association for Molecular Pathology. Genet Med. 2015;17:405–24. 25741868 10.1038/gim.2015.30 PMC4544753

[19] Karczewski KJ, Francioli LC, Tiao G, Cummings BB, Alföldi J, Wang Q, et al. The mutational constraint spectrum quantified from variation in 141,456 humans. Nature. 2020;581:434–43. 32461654 10.1038/s41586-020-2308-7 PMC7334197

[20] Fei H, Wu Y, Wang Y, Zhang J. Exome sequencing and RNA analysis identify two novel CPLANE1 variants causing Joubert syndrome. Mol Genet Genomic Med. 2022;10:e1877. 35092359 10.1002/mgg3.1877 PMC8922956

[21] Leung GKC, Mak CCY, Fung JLF, Wong WHS, Tsang MHY, Yu MHC, et al. Identifying the genetic causes for prenatally diagnosed structural congenital anomalies (SCAs) by whole-exome sequencing (WES). BMC Med Genomics. 2018;11:93. 30359267 10.1186/s12920-018-0409-z PMC6202811

