# Supplementary material for: Uncertain significance and molecular insights of CPLANE1 variants in prenatal diagnosis of Joubert syndrome: a case report
Source: BMC Pregnancy Childbirth. 2024 Dec 26;24:865. doi: 10.1186/s12884-024-07052-3 (PMC11674561; doi:10.1186/s12884-024-07052-3)
Supplement: Supplementary file 1 — Supplementary Material 1 [file 12884_2024_7052_MOESM1_ESM.docx]

**Supplementary Table1. Clinical features and genetic findings of fetuses with *CPLANE1*-related disorders reported previously.**

|  | **Family 1_patient 1** | **Family 2_patient 2** | **Family 3_patient 3** | **Family 4_patient 4** | **Family 5_patient 5** | **Family 5_patient 6** | **Family 6_patient 7** |
| --- | --- | --- | --- | --- | --- | --- | --- |
| Ethnicity | Chinese | France | France | France | France | France | France |
| Obstetric History | Two failed IVFs | NA | NA | Adverse pregnancy ^a^ | Second sibship | Third sibship | NA |
| Gestational age | 24+6 wg | 29 wg | 27 wg | 26 wg | 34 wg | 27 wg | 14 wg |
| Prenatal findings |  |  |  |  |  |  |  |
| CNS malformation | CVH, MTS | HH | HH | NA | NA | Occipital meningocele | Hydrocephalus |
| Digital defects | PD | PD, SD | PD | PD | PD, SD | PD | PD |
| Oral defects | No | No | NA | No | No | No | NA |
| Other abnormalities | NA | Y-shaped metacarpal abnormalities | Y-shaped metacarpal abnormalities | NA | Y-shaped metacarpal abnormalities, aortic coarctation, short femurs | Y-shaped metacarpal abnormalities | Right fibular agenesis, tibial bowing |
| Postmortem examination |  |  |  |  |  |  |  |
| External inspection | NA | PD, SD | Short buccal frenula | Facial dysmorphism | PD, SD | PD, SD | Facial dysmorphism |
| MRI Imaging | NA | NA | NA | NA | NA | NA | NA |
| Autopsy | NA | MTS, 11 pairs of ribs | MTS | MTS | MTS | MTS | MTS |
| Variants  (NM_023073.3) | c.4646A>T/p.E1549V & c.1233C>A/p.Y411*  (compound heterozygous) | c.3550C>T/p.R1184C & c.9121C>T/p.Q3041*  (compound heterozygosis) | c.2377C>T/p.Q793* &  c.8509G>T/p.V2837L  (compound heterozygosis) | c.493delA/p.I165Yfs*17 & c.3380C>T/p.S1127L  (Compound heterozygosis) | c.3380C>T/p.S1127L & c.3859G>C/p.D1287H  (Compound heterozygosis) | c.3380C>T/p.S1127L & c.3859G>C/p.D1287H  (Compound heterozygosis) | c.3859G>C/p.D1287H & c.7476delT/p.R2493Dfs*6  (Compound heterozygosis) |
| DNA sample | Amniotic fluid | Fetal tissue | Fetal tissue | Fetal tissue | Fetal tissue | Fetal tissue | Fetal tissue |
| Procedure test | Trio-WES | Trio-WES | Trio-WES | Trio-WES | Trio-WES | Trio-WES | Single-WES |
| Functional test | No | NA | NA | NA | NA | NA | NA |
| Diagnosis | JS | OFD VI | OFD VI | OFD VI | OFD VI | OFD VI | OFD VI |
| Ref | This report | [1] | [1, 2] | [1] | [1] | [1] | [1] |

**Supplementary Table1. continued**

|  | **Family 7_patient 8** | **Family 8_patient 9** | **Family 8_patient 10** | **Family 9_patient 11** | **Family 10_patient 12** | **Family 10_patient 12** | **Family 11_patient 13** | **Family 12_patient 14** |  |
| --- | --- | --- | --- | --- | --- | --- | --- | --- | --- |
| Ethnicity | France | Saudi Arabia | Saudi Arabia | Chinese | France | France | Italy | Italy |  |
| Obstetric History | NA | First sibship ^b^ | Second sibship ^b^ | A healthy girl | Second sibship | Third sibship | NA | First sibship |  |
| Gestational age | 23 wg | NA | NA | 23+4 wg | 23 wg | 13 wg | 21+6 wg | 21+3 wg |  |
| Prenatal findings |  |  |  |  |  |  |  |  |  |
| CNS malformation | HH, Occipital meningocele | NA | NA | CVH, MTS | Brain anomalies | Brain anomalies | Posterior fossa abnormality | CVH, MTS, HH |  |
| Digital defects | PD | PD, SD | PD, SD | NA | PD | PD | PD | PD |  |
| Oral defects | NA | NA | NA | NA | Cleft lip | Cleft lip | Cleft lip | NA |  |
| Other abnormalities | Arhinencephaly, common  mesentery | NA | NA | NA | NA | NA | Short long bone, enlarged right atrium, ASD | NA |  |
| Postmortem examination |  |  |  |  |  |  |  |  |  |
| External inspection | BF, facial dysmorphism | Low-set ears, clubfeet, cleft palate | Low-set ears, clubfeet, anal atresia | Polydactyly | Facial dysmorphism, cleft lip and palate | Facial dysmorphism, cleft lip and palate | Polydactyly | NA |  |
| Imaging | NA | NA | NA | NA | Tibial hypoplasia | NA | MTS, CVH, HH | NA |  |
| Autopsy | MTS | NA | ABG, DCC, small cystic kidney | MTS | CVH, MTS | NA | No | Posterior fossa abnormalities, Polydactyly |  |
| Variants  (NM_023073.3) | c.6898delC/p.Q2300Rfs*8  & c.7402C>T/p.Q2468* (compound heterozygous) | c.8471-1G>C  (homozygous) | c.8471-1G>C  (homozygous) | c.3599C>T/p.A1200V & c.3857G>A/p.R1286H  (compound heterozygous) | c.3599C>A/p.A1200E & c.6460C>T/p.Q2154*  (compound heterozygous) | c.3599C>A/p.A1200E & c.6460C>T/p.Gln2154*  (compound heterozygous) | c.3921G>A &  c.‐47‐3C>T  (compound heterozygous) | c.5594dupA/p.N1865Kfs*3  & c.3380C>T/p.S1127L  (compound heterozygous) |  |
| DNA sample | Fetal tissue | Fetal tissue | Fetal tissue | Fetal skin | Fetal tissue | Fetal tissue | Fetal tissue | Fetal tissue |  |
| Procedure test | Single-WES | Trio-WES | Trio-WES | Targeted-ES | Targeted-ES | Sanger sequencing | NA | NA |  |
| Functional test | No | No | No | No | No | No | NA | NA | |
| Diagnosis | OFD VI | JS with OFD | JS with OFD | JS with OFD | OFD VI | OFD VI | OFD VI | OFD VI | |
| Ref | [1] | [3] | [3] | [4] | [5] | [5] | [6] | [6] | |

**Supplementary Table1. continued**

|  | **Family 12_patient 15** | **Family 13_patient 16** | **Family 14_patient 17** | **Family 15_patient 18** | **Family 15_patient 19** | **Family 16_patient 20** | **Family 17_patient 21** |
| --- | --- | --- | --- | --- | --- | --- | --- |
| Ethnicity | Italy | Chinese | Chinese | Chinese | Chinese | Chinese | Chinese |
| Obstetric history | Second sibship | NA | NA | Adverse pregnancy ^c^ | Adverse pregnancy ^c^ | Adverse pregnancy ^d^ | NA |
| Gestational age | 16 wg | NA | 19 wg | 18+4 wg | 20 wg | 23+1 wg | NA |
| Prenatal findings |  |  |  |  |  |  |  |
| CNS malformation | Posterior fossa abnormalities | NA | AC, cerebellum dysplasia, Occipital encephalocele | CVH, DWM, hydrocephalus | Occipital encephalocele | Posterior fossa Blake cyst | Widen cerebellar medullary pool |
| Digital defects | PD | NA | NA | NA | NA | PD | PD |
| Oral defects | NA | NA | NA | NA | NA | NA | NA |
| Other abnormalities | NA | NA | Abnormal shape of cranium | Dextrocardia, VSD, and DORV with PAS | IUGR | NA | Renal cyst |
| Postmortem examination |  |  |  |  |  |  |  |
| External inspection | PD, SD | Cleft palate, LT | PD | Normal | NA | NA | NA |
| Imaging | No | NA | NA | NA | NA | NA | NA |
| Autopsy | No | CVH, HH, DWM, etc. | NA | NA | NA | NA | NA |
| Variants  (NM_023073.3) | c.5594dupA/p.N1865Kfs*3  & c.3380C>T/p.S1127L  (compound heterozygous) | c.4459del/p.S1487Vfs*3  c.7534-14G>A/p.E2512Kfs*18  (compound heterozygous) | c.1384_1385insAG &  c.7691-5_7691-4del  (compound heterozygous) | c.7939delC/p.H2647Ifs*51  (homozygous) | c.7939delC/p.H2647Ifs*51  (homozygous) | c.3599C>T/p.A1200V  & c.834+1G>T  (compound heterozygous) | c.2854_2855insCT/p.N952Tfs*13  & c.3599C>T/p.A1200V  (compound heterozygous) |
| DNA sample | Fetal tissue | Fetal tissue | NA | Fetal tissue | Fetal tissue | Umbilical cord and amniotic fluid | NA |
| Procedure test | NA | Trio-WES | Targeted-ES | Trio-WES | Sanger sequencing | Trio-WES | NA |
| Functional test | NA | Yes | Yes | NA | NA | Yes | NA |
| Diagnosis | OFD VI | JS | JS | JS | JS | OFD VI | JS 17 or OFD VI |
| Ref | [6] | [7] | [8] | [9] | [9] | [10] | [11] |

^a^ The first sibship of the case died at 12 weeks of gestation with MTS and left ventricular hypoplasia, without DNA analysis.

^b^ Conceived from a consanguineous union of first cousins.

^c^ Conceived from a consanguineous couple with a history of two early miscarriages and one pregnancy termination due to widened posterior fossa and cerebellar vermis hypoplasia without DNA analysis.

^d^ The first sibship presented with DDC, CVH, and polydactyly at 21+3 weeks of gestation, while the second pregnancy resulted in spontaneous early miscarriages, both without DNA analysis.

Abbreviations: ABG, abnormal brain gyration; AC, arachnoid cysts; ASD, Atrial Septal Defect; BF, additional buccal frenulae; CNS, Central Nervous System; CVH, Cerebellar vermis hypoplasia; DCC, dysplastic corpus callosum; DORV, double outlet right ventricle, DWM, Dandy-Walker malformation; ES, exome sequencing; HH, hypothalamic hamartoma; IUGR, intra-uterine growth restriction; IVF, in vitro fertilization; JS, Joubert syndrome; LT, lobulated tongue; MRI, Magnetic resonance imaging; MTS, molar tooth sign; NA, not available; OFD VI, Orofaciodigital syndrome VI; PAS, pulmonary artery stenosis; PD, Polydactyly; SD, Syndactyly; VSD, ventricular septal defect; WES, whole-exome sequencing; wg, weeks of gestation;

**Reference**

1. Lopez E, Thauvin-Robinet C, Reversade B, Khartoufi NE, Devisme L, Holder M, et al. C5orf42 is the major gene responsible for OFD syndrome type VI. Hum Genet. 2014;133:367–77.

2. Poretti A, Vitiello G, Hennekam RC, Arrigoni F, Bertini E, Borgatti R, et al. Delineation and Diagnostic Criteria of Oral-Facial-Digital Syndrome Type VI. Orphanet J Rare Dis. 2012;7:4.

3. Wentzensen IM, Johnston JJ, Keppler-Noreuil K, Acrich K, David K, Johnson KD, et al. Exome sequencing identifies novel mutations in C5orf42 in patients with Joubert syndrome with oral–facial–digital anomalies. Hum Genome Var. 2015;2:15045.

4. Xiang J, Zhang L, Jiang W, Zhang Q, Wang T, Li H, et al. Prenatal Diagnosis and Genetic Analysis of a Fetus with Joubert Syndrome. Biomed Res Int. 2018;2018:7202168.

5. Bonnard C, Shboul M, Tonekaboni SH, Ng AYJ, Tohari S, Ghosh K, et al. Novel mutations in the ciliopathy-associated gene CPLANE1 (C5orf42) cause OFD syndrome type VI rather than Joubert syndrome. Eur J Med Genet. 2018;61:585–95.

6. Dordoni C, Prefumo F, Iascone M, Pinelli L, Palumbo G, Bondioni MP, et al. Prenatal findings in oral‐facial‐digital syndrome type VI: Report of three cases and literature review. Prenatal Diag. 2019;39:652–5.

7. Fei H, Wu Y, Wang Y, Zhang J. Exome sequencing and RNA analysis identify two novel CPLANE1 variants causing Joubert syndrome. Mol Genet Genomic Med. 2022;10:e1877.

8. Zhu H, Chen W, Ren H, Zhang Y, Niu Y, Wu D, et al. Non-classic splicing mutation in the CPLANE1 (C5orf42) gene cause Joubert syndrome in a fetus with severe craniocerebral dysplasia. Eur J Med Genet. 2021;64:104212.

9. Liu Y, Wang H, Jin X, Shao Q, Pan Q. Molecular Diagnosis and Prenatal Phenotype Analysis of Eight Fetuses With Ciliopathies. Front Genet. 2021;12:705808.

10. Qian W, Liu X, Wang Z, Xu Y, Zhang J, Li H, et al. Whole‐exome sequencing identified novel variants in CPLANE1 that causes oral‐facial‐digital syndrome Ⅵ by inducing primary cilia abnormality. J Cell Mol Med. 2022;26:3213–22.

11. Qin Y, Yao Y, Liu N, Wang B, Liu L, Li H, et al. Prenatal whole-exome sequencing for fetal structural anomalies: a retrospective analysis of 145 Chinese cases. Bmc Med Genomics. 2023;16:262.
